# Classification of different Hepatitis B infected individuals with saturated incidence rate

**DOI:** 10.1186/s40064-016-2706-3

**Published:** 2016-07-15

**Authors:** Tahir Khan, Gul Zaman

**Affiliations:** Department of Mathematics, University of Malakand, Chakdara Dir (Lower), Khyber Pakhtunkhawa, Pakistan

**Keywords:** Hepatitis B model, Stability analysis, Saturated incidence rate, Lypanavo theory, Geometrical approach, Numerical simulation

## Abstract

The role of incidence rate is very important in the study of epidemiological models. In this article, the analysis of an epidemic problem for the transmission dynamic of HBV with saturated incidence rate is presented, which is more generalize than bilinear incidence rate. After formulating the new mathematical model, the threshold quantity reproduction number $R_0$ is investigated by using the well known approach i.e. next generation matrix and investigate the possible equilibriums such as disease free and endemic equilibria. Then for the local and global behavior of the proposed problem, the local asymptotic stability analysis as well as global asymptotic stability analysis are proved. To prove the global asymptotic stability at disease free equilibrium, the classic Lyapunov function theory is considered. Similarly to show global asymptotic stability at endemic equilibria, the geometrical approach is used, which is the generalization of Lyapunov theory. Finally, numeric of the proposed problem are carried out to show the feasibility of the obtained results and the role of saturated incidence rate.

## Background

Mathematical modeling is a power full tool to study the dynamic of different diseases in the real world phenomena (Zaman et al. 2008, 2009; Mann and Roberts 2011; Zhao et al. 2000). Mathematician and Biologist used different widespread models to understand the spreading of transmittible maladies in the population. Hepatitis B is a globally health problem and a prominent root of expiry in the world. Billion of populates are septic with Hepatitis B virus contamination. Throughout the world 2 billion individuals have been sick with Hepatitis B virus and around 2 million individuals live chronically infected with Hepatitis B. Every year nearly 780,000 individuals expires due to chronic or acute Hepatitis B virus infection (WHO 2014). Thus from the evidence it is clear that Hepatitis B virus infection is responsible for about 80 % of the primary liver cancer.

The transmission of Hepatitis B virus are very common and can be transmitted form one individual to another individual on both ways horizontally and vertically, such as transmission of blood, semen, vaginal secretions (Lavanchy 2004; Lok et al. 2001; McMahon 2005), unprotected sexual contact, sharing of razers, blades or tooth brushes (Chang 2007) and infected mother to her/his child during the time of birth, respectively. However Hepatitis B virus cannot be transmitted through water, food, hugging, kissing and causal contact such as in the work place, school etc. (McMahon 2005). The mode of transmission of Hepatitis B virus and HIV are the same, but Hepatitis B virus is fifty to hundreds time more infectious (Thornley et al. 2008; Ali and Zaman 2016).

Infection of Hepatitis B has different phases, such as acute Hepatitis, chronic and carrier Hepatitis. Acute infection of Hepatitis B denoted the first 6 months subsequently exposed some one to Hepatitis B virus. Herein the resistant organism is frequently capable to flawless the infection from the physique then the body must be recovered in a few months complectly for some one, but for the remaining infection grow and leads to more serious stage called chronic stage or life long sickness. Chronic stage of Hepatitis B raises to the infection, that happens when Hepatitis B virus remain in an individual body and over period, the contagion can develop stern healthiness problem. Individuals with carrier Hepatitis frequently have no antiquity of acute sickness, but it can cause liver damaging which become the cause liver damaging and could also grow to liver tumor Mann and Roberts (2011).

Numerous of authors have premeditated different widespread models, which label the dynamic of communicable diseases (Zou et al. 2010; Thornley et al. 2008; Mann and Roberts 2011). The incidence rate is one of the key concept and theatres an important role in the field of mathematical modeling. Bilinear occurrence rate $\beta SI$ frequently recycled in various epidemic problems (Fan et al. 2001; Li and Ma 2002; Zou et al. 2010), where $\beta$ represents the contact rate, *S* represents susceptible individuals and *I* represents the infectious individuals. For the first time Capasso and Serio introduced the saturated incidence rate $\frac{\beta SI}{1+\alpha I},$ which is the generalized form of bilinear incidence rate (Capasso and Serio 1978). This occurrence rate is additional sensible than the bilinear occurrence rate, especially in the case of sexually transmittible diseases, since it embraces the negotiating alteration and swarming influence of the virulent individuals and inhibits the unboundedness of the interaction rate by indicating appropriate parameters, which was recycled in numerous of epidemic problems (Kar and Jana 2013; Gomes et al. 2005; Liu and Yang 2005).

In this article, we present an epidemic problem for the transmission dynamic of Hepatitis B virus with saturated incidence rate, which is the modified version of Zou et al. (2010). After developing the new model, we find the basic reproduction number by using the well known approach i.i. next generation matrix (NGM) approach. Furthermore, we investigate the possible equilibriums i.e. disease free and endemic equilibria and show the local asymptotic stability as well as global asymptotic stability at both equilibriums. For the local asymptotic stability, we use linearization and Routh-Hurwitz criteria, while to discuss the global stability, we then use the classic Lypanavo function theory and geometrical approach. Finally the numerical simulation are achieved by exhausting Runge-Kutta method of order fourth scheme to show the feasibility of the obtained results and the role of saturated incidence rate.

The organization of the paper is as follows. In the second section, we presents the proposed model and studied its different analysis, including basic reproduction number and equilibriums. In section “Stability analysis”, we study the stability analysis and prove the local as well as global stability. Section “Numerical analysis” is devoted to numerical simulation and discussion. Finally a brief conclusion is presented in section “Conclusion”.

## Mathematical model and its analysis

Herein this section, we presents a Hepatitis B virus transmission epidemic model. For this, we split the entire populace into seven epidemiological subclasses, susceptible *S*(*t*), latent *L*(*t*), acute infected *A*(*t*), chronic infected individuals *B*(*t*), carrier individuals *C*(*t*), recovered with permanent immunity *R*(*t*) and vaccinated *V*(*t*). Thus the compartmental mathematical model can be represented by the succeeding system of seven ordinary differential equations,

1 $$\begin{aligned} \frac{dS(t)}{dt} & = b\xi (1-\eta C(t))+\phi V(t)-\frac{\beta S(t)A(t)}{1+\alpha C(t)} -\frac{\gamma \beta S(t)B(t)}{1+\alpha C(t)}-\frac{\zeta \beta S(t)C(t)}{1+\alpha C(t)}-(\mu _0+v)S(t),\nonumber \\ \frac{dL(t)}{dt} & = \frac{\beta S(t)A(t)}{1+\alpha C(t)}+\frac{\gamma \beta S(t)B(t)}{1+\alpha C(t)}+\frac{\zeta \beta S(t)C(t)}{1+\alpha C(t)}-(\sigma +\mu _0)L(t),\nonumber \\ \frac{dA(t)}{dt}& = \sigma L(t)-(\mu _0+\gamma _1+\psi )A(t),\nonumber \\ \frac{dB(t)}{dt}& = p\gamma _1A(t)-(\mu _0+\mu _1+\gamma _2)B(t),\nonumber \\ \frac{dC(t)}{dt}& = b \xi \eta C(t)+(1-p)\gamma _1A(t)-(\mu _0+\mu _2+\gamma _3)C(t),\nonumber \\ \frac{dR(t)}{dt}& = \psi A(t)+\gamma _2 B(t)+\gamma _3 C(t)-\mu _0R(t),\nonumber \\ \frac{dV(t)}{dt}& = b(1-\xi )+v S(t)-(\mu _0+\phi )V(t). \end{aligned}$$

Here, *b* represents the birth rate, $$\xi$$ represents the birth rate without successful vaccination, $$\eta$$ represents the proportion of prenatally infected individuals, $$\phi$$ represents the rate of waning vaccine induced immunity, $$\beta$$ represents the transmission rate from susceptible to infected, $$\gamma$$, $$\zeta$$ represent the reduced transmission rate of chronic and carrier Hepatitis B infected individuals, respectively. $$\mu _0$$ represents the death rate, which occur naturally, *v* represents the vaccination rate, $$\sigma$$ represents the moving rate from latent class to acute class, $$\gamma _1$$ represents the moving rate from acute to chronic and carrier, $$\gamma _2$$ represents the moving rate of chronic carrier to immune, $$\gamma _3$$ represents the moving rate of carrier to immune, $$\mu _1,$$$$\mu _2$$ represents the death rate, which occur from the Hepatitis B and *p* represents the average probability of those individuals, who fails to recovers in acute stage and goes to chronic carrier.

Let “$N(t)$” represents the entire populace, such that, $N(t)=S(t)+L(t)+A(t)+B(t)+C(t)+R(t)+V(t).$ The initial conditions with the proposed model, make sure that, $N(t)\ge 0.$ Hence the entire populace “$N(t)$” is bounded and persist positive for $t>0.$ Now the time derivative of *N*(*t*) yields

2 $$\begin{aligned} \frac{dN(t)}{dt}=b-\mu _0N(t)-\mu _1B(t)-\mu _2C(t), \end{aligned}$$

which implies that, for large time *t*,  that is $$t\rightarrow \infty ,$$$$N(t)\le \frac{b}{\mu _0}.$$ Thus the feasible region for our proposed model is

3 $$\begin{aligned} \Omega =\left\{ (S(t),L(t),A(t),B(t),C(t),R(t),V(t))\in R_{+}^7,N\le \frac{b}{\mu _0}\right\} . \end{aligned}$$

Therefore, we study the dynamic of our proposed model (1) in the feasible region $\Omega ,$ which is an attracting set.

For obtaining the Jacobian matrix of our model, we take the reduced system, because *R* dose not appears explicitly in all others classes, so the Jacobian matrix of the reduced system (with out *R*) becomes

4 $$\begin{aligned} J=\left( \begin{array}{cccccc} -a_{11} &{} 0 &{} -a_{13} &{} -a_{14} &{} -a_{15} &{} \phi \\ a_{21} &{} -a_{22} &{} a_{23} &{} a_{24} &{} a_{25} &{} 0\\ 0 &{} \sigma &{} -a_{33} &{} 0 &{} 0 &{} 0\\ 0 &{} 0 &{} p\gamma _1 &{} a_{44} &{} 0 &{} 0\\ 0 &{} 0 &{} a_{53} &{} 0 &{} -a_{55} &{} 0\\ v &{} 0 &{} 0 &{} 0 &{} 0 &{} -a_{66} \end{array} \right) , \end{aligned}$$

where

$$\begin{aligned} a_{11} &= \mu _0+v+\frac{1}{1+\alpha C}(\beta A+\gamma \beta B+\zeta \beta C),~a_{13}=a_{23}=\frac{\beta S}{1+\alpha C},~a_{14}=a_{24}=\frac{\gamma \beta B}{1+\alpha C},\\ a_{15} &= \frac{1}{(1+\alpha C)^2}(\zeta \beta S-\alpha \beta S A-\alpha \gamma \beta S B),~a_{21}=a_{11}-(\mu _0+v),~a_{22}=\sigma +\mu _0,\\ a_{33} &= \mu _0+\gamma _1+\psi , ~a_{44}=\mu _0+\mu _1+\gamma _2,~a_{55}=b\xi \eta -(\mu _0+\mu _2+\gamma _3),~a_{66}=\mu _0+\phi . \end{aligned}$$

### Basic reproduction number

In epidemiological models the inception capacity $R_0$ is called the basic reproduction number is a key concept, which characterizes the anticipated average amount of new contaminations created directly and circuitously by a solitary infectious, when familiarized into a entirely susceptible populace (Anderson and May 1991; van den Driessche and Watmough 2002). To find the this quantity for our proposed model (1), we use the method of Van Den Driessche and Watmough (2008). Let $\chi =(L(t),A(t),B(t),C(t)),$ so from the model (1), we have

5 $$\begin{aligned} \frac{d\chi }{dt}=\bar{F}-\bar{V}. \end{aligned}$$

In Eq. (5), $\bar{F}$ and $\bar{V}$ are define as

$$\begin{aligned} \bar{F}=\left( \begin{array}{c} \beta S(t)A(t)+\gamma \beta S(t)B(t)+\zeta \beta S(t)C(t) \\ 0 \\ 0 \\ 0 \\ \end{array} \right) ,\bar{V}=\left( \begin{array}{c} r_1\\ r_2\\ r_3\\ r_4\\ \end{array} \right) , \end{aligned}$$

where $$r_1=(\sigma +\mu _0)L(t),$$$$r_2=(\mu _0+\gamma _1+\psi )A(t)-\sigma L(t),$$$$r_3=(\mu _0+\mu _1+\gamma _2)B(t)-p\gamma _1A(t)$$ and $$r_4=(\mu _0+\mu _2+\gamma _3)C(t)-b\xi \eta C(t)-(1-p)\gamma _1 A(t).$$ Now we find the Jacobian of $$\bar{F}$$ and $$\bar{V}$$ at diseases free equilibrium $$F_0,$$ thus we have

$$\begin{aligned} F =\left( \begin{array}{cccc} 0 &\quad \beta S_0 &\quad \gamma \beta S_0 &\quad \zeta \beta S_0 \\ 0 &\quad 0 &\quad 0 &\quad 0 \\ 0 &\quad 0 &\quad 0 &\quad 0 \\ 0 &\quad 0 &\quad 0 &\quad 0 \\ \end{array} \right) ,V =\left( \begin{array}{cccc} a_{11} &\quad 0 &\quad 0 &\quad 0 \\ -\sigma &\quad a_{22} &\quad 0 &\quad 0 \\ 0 &\quad -a_{32} &\quad a_{33} &\quad 0 \\ 0 &\quad -a_{41} &\quad 0 &\quad a_{44} \\ \end{array} \right) , \end{aligned}$$

where $$a_{11}=\sigma +\mu _0,$$$$a_{22}=\mu _0+\gamma _1+\psi ,$$$$a_{32}=p\gamma _1,$$$$a_{33}=\mu _0+\mu _1+\gamma _2,$$$$a_{41}=(1-p)\gamma _1$$ and $$a_{44}=\mu _0+\mu _0+\gamma _3-b\xi \eta .$$ Thus $$R_0$$ is the spectral radius of $$\bar{K}=F V^{-1},$$ that is $$R_0=\rho (FV^{-1} )$$. So the basic reproduction number $$R_0$$ for our proposed model (1) becomes

6 $$\begin{aligned} R_0=\bar{R_1}+\bar{R_2}+\bar{R_3}, \end{aligned}$$

where

$$\begin{aligned} \bar{R_1} &= \frac{\sigma \beta S_0}{(\sigma +\mu _0)(\gamma _1+\psi +\mu _0)},\\ \bar{R_2} &= \frac{\sigma \beta \gamma \gamma _1p S_0 }{(\sigma +\mu _0)(\gamma _1+\psi +\mu _0)(\gamma _2+\mu _0+\mu _1)},\\ \bar{R_3} &= \frac{\sigma \beta \zeta \gamma _1 (1-p) S_0}{(\sigma +\mu _0)(\gamma _1+\psi +\mu _0)(\gamma _3+\mu _0+\mu _2-b \xi \eta )} . \end{aligned}$$

### Equilibrium analysis

To study the dynamics of our proposed model (1), we need to find the equilibriums, thus the disease free equilibrium of the model (1) is denoted by $F_0$ and define as $F_0=(S_0,0,0,0,0,0,V_0),$ where

7 $$\begin{aligned} S_0=\frac{b(\phi +\mu _0\xi )}{\mu _0(\mu _0+v+\phi )},~V_0=\frac{b(\mu _0+v-\mu _0\xi )}{\mu _0(\mu _0+v+\phi )}. \end{aligned}$$

Similarly endemic equilibrium is denoted by $F_1$ and define as $F_1=(S_1,L_1,A_1,C_1,R_1,V_1),$ where

8 $$\begin{aligned} \nonumber S_1 & = S_0-\frac{1}{q_3q_5q_7}b\xi \eta \gamma _1(1-p)q_6L_1-\frac{1}{q_7}q_2q_6L_1,\\ \nonumber L_1& = \frac{q_2q^2_3q_4q^2_5q_7(R_0-1)}{\sigma (w_1(1-p) +\beta (1+p\gamma _1)q_2q_3q_4q^2_5q_6)},\\ \nonumber A_1 &= \frac{q_2q_3q_4q^2_5q_7(R_0-1)}{w_1(1-p) +\beta (1+p\gamma _1)q_2q_3q_4q^2_5q_6},\\ \nonumber B_1 &= \frac{q_2q_3q^2_5q_7p\gamma _1(R_0-1)}{w_1(1-p) +\beta (1+p\gamma _1)q_2q_3q_4q^2_5q_6},\\ C_1 &= \frac{q_2q_3q_4q_5q_7(1-p)(R_0-1)}{w_1(1-p) +\beta (1+p\gamma _1)q_2q_3q_4q^2_5q_6},\\ \nonumber R_1 & = \frac{1}{\mu _0}(\psi A_1+\gamma _2B_1+\gamma _3C_1),\\ \nonumber V_1 &= \frac{1}{q_6}(b(1-\xi )+vS_1), \end{aligned}$$

where $$q_1=\mu _0+v,$$$$q_2=\sigma +\mu _0,$$$$q_3=\mu _0+\gamma _1+\psi ,$$$$q_4=\mu _0+\mu _1+\gamma _2,$$$$q_5=\mu _0+\mu _2+\gamma _3-b\xi \eta ,$$$$q_6=\mu _0+\phi ,$$$$q_7=\mu _0(\mu _0+v+\phi )$$ and $$w_1=\sigma \beta b \xi \eta \gamma _1 q_5(q_4+\gamma _1pq_6+\gamma _1q_2q_3q_4q_5(\alpha q_7+\beta q_6)+\alpha \beta b\xi \eta \gamma ^2_1q_4q_6)$$.

## Stability analysis

Herein this section, we want to study asymptotic stability of our proposed model at both equilibria. So first, we prove the local stability of our model at illness free and endemic equilibrium, then the global asymptotic stability. For the local stability, we use the linearization method, but for global dynamic stability, we want to use both the Lypanavo function theory and geometrical approach.

### Local stability analysis

For the local dynamic of the proposed model at disease free and endemic equilibrium points, we state and prove the following results.

#### **Theorem 1**

*If*$R_0<1$, *then the model* (1) *is locally asymptotically stable at disease free equilibrium point*$F_0$*and if*$R_0>1$, *then it is the unstable saddle point*.

#### *Proof*

The characteristic equation of the Jacobian matrix *J* (4) at disease free equilibrium $F_0$ becomes

9 $$\begin{aligned} P(\lambda )=(\lambda +\mu _0)(\lambda +\mu _0+v+\phi )(\lambda ^4+a_1\lambda ^3+a_2\lambda ^2+a_3\lambda +a_4)=0, \end{aligned}$$

where

$$\begin{aligned} a_1 & = a_{22}+a_{33}+a_{44}+a_{55},\\ a_2 & = a_{22}a_{33}(1-R_1)+a_{44}a_{55}+a_{55}a_{33}+a_{33}a_{44}+a_{22}a_{44}+a_{22}a_{55},\\ a_3 & = a_{55}a_{33}a_{22}(1-R_1)+a_{33}a_{44}a_{55}\left( 1-\frac{a_{22}}{a_{55}}R_2\right) +a_{22}a_{44}a_{55}(1-R_2)+a_{22}a_{33}a_{44}(1-R_1),\\ a_4 & = \sigma \gamma \beta p \gamma _1S_0a_{55}+\sigma \zeta p\gamma _1 \beta S_0a_{44}+a_{22}a_{33}a_{55}-\sigma \beta S_0a_{44}b_{55}-\sigma \zeta \beta \gamma _1S_0a_{44}. \end{aligned}$$

If $R_0<1,$ we have $0<\bar{R_i}<1,$ for $i=1,2,3.$ So $a_j>0,$ for $j=1,2,3,4,$ and also it is easy to show that, $a_1a_2a_3>a^{2}_3+a^2_2a_4.$ Therefore Routh-Herwitz criteria is satisfied, that is all the roots of the characteristic polynomial $P(\lambda )$ have negative real parts, which ensure that the disease free equilibrium point $F_0$ is stable, while for $R_0>1,$ the system have both negative and positive eigenvalues, which states that the disease free equilibrium point is a saddle point, always unstable.

#### **Theorem 2**

*If*$R_0>1$, *then the model* (1) *is locally asymptotically stable at endemic equilibrium point*$F_1$*and if*$R_0<1$, *then it is the unstable*.

#### *Proof*

Using the elementary row operation, reducing the Jacobian matrix at Eq. 4 around the endemic equilibrium $F_1$, we get the following echelon form

10 $$\begin{aligned} J_1=\left( \begin{array}{cccccc} -l_{11} &{} 0 &{} -l_{12} &{} -l_{13} &{} -l_{14} &{} \phi \\ 0 &{} -l_{22} &{} l_{23} &{} l_{24} &{} l_{25} &{} \phi l_{21} \\ 0 &{} 0 &{} l_{33} &{} l_{34} &{} 0 &{} \sigma \phi l_{21} \\ 0 &{} 0 &{} 0 &{} l_{44} &{} l_{45} &{} -\sigma \phi l_{21} \\ 0 &{} 0 &{} 0 &{} 0 &{} l_{44} &{} \sigma \phi l_{21} \\ 0 &{} 0 &{} 0 &{} 0 &{} 0 &{} l_{66} \\ \end{array} \right) , \end{aligned}$$

where $$l_{11}=a_{11},$$$$l_{12}=a_{12},$$$$l_{13}=a_{13},$$$$l_{15}=a_{15},$$$$l_{22}=a_{11}a_{22},$$$$l_{23}=a_{23}(a_{11}-a_{12}),$$$$l_{24}=a_{14}(a_{11}-a_{21}),$$$$l_{25}=a_{15}(a_{11}-a_{21}),$$$$l_{33}=-a_{11}a_{22}a_{33}+\sigma a_{13}(a_{11}-a_{21}),$$$$l_{34}=\sigma a_{14}(a_{11}-a_{21}),$$$$l_{35}=\sigma a_{14}(a_{11}-a_{21}),$$$$l_{44}=-\frac{1}{p\gamma _1}\sigma a_{13}a_{44}(a_{11}a_{22}a_{33}+a_{11}-a_{21}),$$$$l_{45}=a_{14}(a_{11}-a_{21}),$$$$l_{45}=a_{14}(a_{11}-a_{21}),$$$$l_{55}=l_{44}+\sigma a_{14}(a_{11}-a_{21})$$ and $$l_{66}=l_{44}-\frac{\sigma \phi v a_{14} a_{44}a_{53}(\mu _0+v)}{\beta p\gamma _1\zeta a_{44}+\beta a_{45}(p\gamma \gamma _1+a_{44}))}.$$

Thus after some simplification and little re-arrangement the eigenvalues of the Jacobian matrix $J_1$ are given by

$$\begin{aligned} \nonumber \lambda _1 & = -a_{11},~\lambda _2=-a_{22},~\lambda _3=-a_{11}a_{22}a_{33}+\sigma a_{13}(a_{11}-a_{21}),\\ \lambda _4 & = -\frac{1}{p\gamma _1}\sigma a_{13}a_{44}(a_{11}a_{22}a_{33}+a_{11}-a_{21}),\\ \lambda _5 & = -l_{44}+\sigma a_{14}(a_{11}-a_{21}),\\ \lambda _6 & = l_{44}-\frac{\sigma \phi v a_{14} a_{44}a_{53}(\mu _0+v)}{\beta p\gamma _1\zeta a_{44}+\beta a_{45}(p\gamma \gamma _1+a_{44}))}. \end{aligned}$$

Obviously $$\lambda _1,$$$$\lambda _2,$$$$\lambda _4,$$$$\lambda _6$$ have negative real parts and $$\lambda _3,$$$$\lambda _5$$ are negative, if $$a_{22}a_{33}>\sigma \beta S_1$$ and $$a_{44}S_1>\gamma B_1.$$ Therefore for $$R_0>1,$$ the model (1) is locally asymptotically stable at endemic equilibrium point $$F_1,$$ if $$a_{22}a_{33}>\sigma \beta S_1$$ and $$a_{44}S_1>\gamma B_1.$$

### Global stability analysis

To show the global stability of the proposed model, using Lypanavo function theory and geometrical approach. Here, we want to prove the global stability of disease free equilibrium point $F_0$ by using Lypanavo function theory, while to prove the global stability at endemic equilibrium point $F_1,$ we use geometrical approach. Before to presents the stability results, the following important lemma due to Li and Muldowney (1996) is quoted. So, we obtain the condition at which the proposed model is globally asymptotically stable.

#### **Lemma 1**

*If the system*$$\dot{y}=g(y),$$$$g:D\rightarrow R^{n}$$*containing a unique equilibrium of the form*$$y^*$$*and there is a compact absorbing set, then this system is globally asymptotically stable around that equilibrium, if there exist a functionD*(*y*) *and a Lozinskii measure*$$\ell ,$$*such that*$$\lim _{t\rightarrow \infty }supsup\frac{1}{t}\int ^t_0\ell (B)dt<0.$$

Thus regarding the global stability of the proposed model at disease free and endemic equilibrium points, we have the following results.

#### **Theorem 3**

*If*$R_0<1$, *then the model* (1) *is globally asymptotically stable at disease free equilibrium point*$F_0$*and unstable otherwise*.

#### *Proof*

To show the global stability at disease free equilibrium point $F_0$, we use Lypanavo function theory, so consider the following Lypanavo function, such that

11 $$\begin{aligned} \nonumber F(t) & = \frac{1}{2}[(S-S_0)+L(t)+A(t)+B(t)+C(t)+R(t)+(V-V_0)]^2+\lambda _1S(t)+\lambda _2L(t)\\&\quad +\lambda _3A(t)+\lambda _4B(t)+\lambda _5V(t), \end{aligned}$$

where $\lambda _i$ for $i=1,2,3,4,5$ are some positive constants, which will be chosen latter. After differentiating *F*(*t*) with respect to time, we get

12 $$\begin{aligned} \nonumber \frac{dF}{dt} & = [(S-S_0)+L(t)+A(t)+B(t)+C(t)+R(t)+(V-V_0)](b-\mu _0N(t)-\mu _1B(t))\\& \quad+\lambda _1\frac{dS}{dt}+\lambda _2\frac{dL}{dt}+\lambda _3\frac{dA}{dt}+\lambda _4\frac{dB}{dt}+\lambda _5\frac{dV}{dt}. \end{aligned}$$

Choosing the positive parameters $\lambda _i=q_2q_4,$ for $i=1,2,3,5$ and $\lambda _4=\sigma \beta \gamma S_0$ along the solution of the model (1), we get

13 $$\begin{aligned} \nonumber \frac{dF}{dt} & = [(S-S_0)+L(t)+A(t)+B(t)+C(t)+R(t)+(V-V_0)](b-\mu _0N(t)-\mu _1B(t))\\& \quad -q_2q_4(b\xi \eta C(t)-\mu _0L(t))-q_2q_3q_4(1-\bar{R_2})A(t)-\sigma \beta \gamma \gamma _1pS_0q_4B(t)\\ \nonumber& \quad +(b-\mu _0S(t)-\mu _0V(t))q_2q_4. \end{aligned}$$

Since $N(t)\le \frac{b}{\mu _0},$ which implies that $S(t)\le \frac{b}{\mu _0}.$ Thus using $S_0+V_0=\frac{b}{\mu _0}$ and $S(t)\le \frac{b}{\mu _0}$ in Eq. (13), we obtain

14 $$\begin{aligned} \nonumber \frac{dF}{dt}\le & {} -[(S-S_0)+L(t)+A(t)+B(t)+C(t)+R(t)+(V-V_0)](\mu _0(N(t)-S(t))\\&+\mu _1B(t))-q_2q_4(b\xi \eta C(t)+\mu _0L(t))-q_2q_3q_4(1-\bar{R_2})A(t)-\sigma \beta \gamma \gamma _1pS_0q_4B(t)\\ \nonumber&-\mu _0((S(t)+V(t))-(S_0+V_0)q_2q_4. \end{aligned}$$

If $$R_0<1,$$ we have $$0<\bar{R_i}<1,$$ for $$i=1,2,3,$$ therefore $$\frac{dF}{dt}$$ is negative, Also $$\frac{dF}{dt}=0,$$ if $$S=S_0,$$$$L=L_0,$$$$A=A_0,$$$$B=B_0,$$$$C=C_0,$$$$R=R_0$$ and $$V=V_0,$$ thus the largest compact invariant set in $$\Omega$$ is the singleton set $$\{F_0\},$$ so “LaSalle’s invariant principle” implies that, the illness free equilibrium point $$F_0$$ is globally asymptotically stable.

#### **Theorem 4**

*If*$R_0>1$, *then the model* (1) *is globally asymptotically stable at endemic equilibrium point*$F_1$.

#### *Proof*

Let $J_2$ and $J_{3}$ be the Jacobian matrix and second additive compound matrix containing only the first three equation of the model (1), then we have

15 $$\begin{aligned} J_2=\left( \begin{array}{ccc} -a_{11} &{} 0 &{} -a_{13} \\ a_{21} &{} a_{22} &{} a_{23} \\ 0 &{} \sigma &{} -a_{33} \\ \end{array} \right) ,~~J_{3}=\left( \begin{array}{ccc} -(a_{11}+a_{22}) &{} a_{23} &{} -a_{13}\\ a_{32} &{} -(a_{11}+a_{33}) &{} a_{12}\\ -a_{31} &{} a_{21} &{} -(a_{22}+a_{33})\\ \end{array} \right) . \end{aligned}$$

Let us consider the function $P(\chi )=P(S,L,A)=diag\left\{ \frac{S}{L},\frac{S}{L},\frac{S}{L}\right\} ,$ which implies that $P^{-1}(\chi )=diag\left\{ \frac{L}{S},\frac{L}{S},\frac{L}{S}\right\} ,$ then taking the time derivative, that is $P_f(\chi )$, we get

16 $$\begin{aligned} P_f(\chi )=diag\left\{ \frac{\dot{S}}{S}-\frac{S\dot{L}}{L^{2}},\frac{\dot{S}}{S}-\frac{S\dot{L}}{L^{2}},\frac{\dot{S}}{S}-\frac{S\dot{L}}{L^{2}}\right\} . \end{aligned}$$

Now $P_fP^{-1}=diag\left\{ \frac{\dot{S}}{S}-\frac{\dot{L}}{L},\frac{\dot{S}}{S}-\frac{\dot{L}}{L},\frac{\dot{S}}{S}-\frac{\dot{L}}{L}\right\}$ and $PJ_{3}2P^{-1}=J_{3}.$ Thus we take $B=P_fP^{-1}+PJ_{3}P^{-1}$, which can be written as

17 $$\begin{aligned} B=\left( \begin{array}{cc} B_{11} &{} B_{12}\\ B_{21} &{} B_{22} \end{array} \right) , \end{aligned}$$

where

$$\begin{aligned} B_{11} & = \frac{\dot{S}}{S}-\frac{\dot{L}}{L}-\frac{\beta A(t)}{1+\alpha C(t)}-\frac{\gamma \beta B(t)}{1+\alpha C(t)}-\frac{\zeta B C(t)}{1+\alpha C(t)}-2\mu _0-v-\sigma ,\\ B_{12} & = \left( \begin{array}{cc} \frac{\beta S(t)}{1+\alpha C(t)}&{} \frac{\beta S(t)}{1+\alpha C(t)}\\ \end{array} \right) , ~B_{21}=\left( \begin{array}{c} \sigma \\ 0 \\ \end{array} \right) ,~ B_{22}=\left( \begin{array}{cc} x_{11} &{} 0 \\ x_{21}&{} x_{22} \\ \end{array} \right) . \end{aligned}$$

In the above matrix $$x_{11}=\frac{\dot{S}}{S}-\frac{\dot{L}}{L}-\frac{\beta A(t)}{1+\alpha C(t)}-\frac{\gamma \beta B(t)}{1+\alpha C(t)}-\frac{\zeta \beta C(t)}{1+\alpha C(t)}-2\mu _0-v-\gamma _1-\psi ,$$$$x_{21}=\frac{\beta A(t)}{1+\alpha C(t)}+\frac{\gamma \beta B(t)}{1+\alpha C(t)}+\frac{\zeta \beta C(t)}{1+\alpha C(t)}$$ and $$x_{22}=\frac{\dot{S}}{S}-\frac{\dot{L}}{L}-2\mu _0-\sigma -\gamma _1-\psi .$$ Let $$(b_1,b_2,b_3)$$ be a vector in $$R^{3}$$ and its norm $$\Vert .\Vert$$ defined by

18 $$\begin{aligned} \Vert b_1,b_2,b_3\Vert =max\{\Vert b_1\Vert ,\Vert b_2\Vert +\Vert b_3\Vert \}. \end{aligned}$$

Now we take the Lozinski measure $\ell (B)$ with respect to the above norm described by Martin (1974), that is $\ell (B)\le sup\{g_1,g_2\}=sup\{\ell (B_{11})+\Vert B_{12}\Vert ,\ell (B_{22})+\Vert B_{21}\Vert \},$ where $g_i=\ell (B_{ii})+\Vert B_{ij}\Vert$ for $i=1,2$ and $i\ne j,$ which implies that

19 $$\begin{aligned} g_1=\ell (B_{11})+\Vert B_{12}\Vert ,~~g_2=\ell (B_{22})+\Vert B_{21}\Vert , \end{aligned}$$

where $\ell (B_{11})=\frac{\dot{S}}{S}-\frac{\dot{L}}{L}-\frac{\beta A(t)}{1+\alpha C(t)}-\frac{\gamma \beta B(t)}{1+\alpha C(t)}-\frac{\zeta B C(t)}{1+\alpha C(t)}-2\mu _0-v-\sigma$, $\Vert B_{12}\Vert =\frac{\beta S(t)}{1+\alpha C(t)}$, $\ell (B_{22})=max\left\{ \frac{\dot{S}}{S}-\frac{\dot{L}}{L}-2\mu _0-v-\gamma _1-\psi ,\frac{\dot{S}}{S}-\frac{\dot{L}}{L}-2\mu _0-\sigma -\gamma _1-\psi \right\} = \frac{\dot{S}}{S}-\frac{\dot{L}}{L}-2\mu _0-\gamma _1-\psi -min\{v,\sigma \}$ and $\Vert B_{21}\Vert =max\{\sigma ,0\}=\sigma .$ Therefore $g_1$ and $g_2$ becomes

$$\begin{aligned} g_1 & = \frac{\dot{S}}{S}-\frac{\dot{L}}{L}-\frac{\beta A(t)}{1+\alpha C(t)}-\frac{\gamma \beta B(t)}{1+\alpha C(t)}-\frac{\zeta \beta C(t)}{1+\alpha C(t)}-2\mu _0-min\{v,\sigma \}+\frac{\beta S(t)}{1+\alpha C(t)},\\ g_2 & = \frac{\dot{S}}{S}-\frac{\dot{L}}{L}-2\mu _0-\sigma -\gamma _1-\psi . \end{aligned}$$

Thus, we can write $g_1\le \frac{\dot{S}}{S}-2\mu _0-min\{v,\sigma \}$ and $g_2\le \frac{\dot{S}}{S}-2\mu _0-\gamma _1-\psi ,$ which implies that $\ell (B)\le \left\{ \frac{\dot{S}}{S}-2\mu _0-min\{min\{v,\sigma \},\gamma _1-\psi \}\right\} .$ Hence $\ell (B)\le \frac{\dot{S}}{S}-2\mu _0.$ Now integrating the Lozinski measure $\ell (B)$ with respect to *t* in the interval [0, *t*] and taking $lim_{t\rightarrow \infty },$ we obtain

20 $$\begin{aligned} lim_{t\rightarrow \infty }sup~sup \frac{1}{t} \int ^{t}_{0}\ell (B)dS\le -2\mu _0<0. \end{aligned}$$

So finally, we can write

21 $$\begin{aligned} \bar{q}=lim_{t\rightarrow \infty }sup~sup \frac{1}{t} \int ^{t}_{0}\ell (B)dS<0. \end{aligned}$$

Thus the subsystem, which containing first three equations of the model (1) is globally asymptotically stable around its interior equilibrium $(S_1,L_1,A_1).$ Now consider the subsystem of the model (1), such that

22 $$\begin{aligned} \nonumber \frac{dB(t)}{dt} & = p\gamma _1A(t)-(\mu _0+\mu _1+\gamma _2)B(t),\\ \nonumber \frac{dC(t)}{dt} & = b \xi \eta C(t)+(1-p)\gamma _1A(t)-(\mu _0+\mu _2+\gamma _3)C(t),\\ \frac{dR(t)}{dt} & = \psi A(t)+\gamma _2 B(t)+\gamma _3 C(t)-\mu _0R(t),\\ \nonumber \frac{dV(t)}{dt} & = b(1-\xi )+v S(t)-(\mu _0+\phi )V(t). \end{aligned}$$

Taking the limit system of the model (22), we get

23 $$\begin{aligned} \nonumber \frac{dB(t)}{dt} & = p\gamma _1A_1(t)-(\mu _0+\mu _1+\gamma _2)B(t),\\ \nonumber \frac{dC(t)}{dt} & = b \xi \eta C(t)+(1-p)\gamma _1A_1(t)-(\mu _0+\mu _2+\gamma _3)C(t),\\ \frac{dR(t)}{dt} & = \psi A_1(t)+\gamma _2 B_1(t)+\gamma _3 C_1(t)-\mu _0R(t),\\ \nonumber \frac{dV(t)}{dt} & = b(1-\xi )+v S_1(t)-(\mu _0+\phi )V(t). \end{aligned}$$

Solving system (38) and using the initial conditions *B*(0), *C*(0),  *R*(0) and *V*(0). So for large time *t* that is $$t\rightarrow \infty$$, $$B(t)\rightarrow B_1,$$$$C(t)\rightarrow C_1,$$$$R(t)\rightarrow R_1$$ and $$V(t)\rightarrow V_1,$$ which is sufficient to prove that the endemic equilibrium point $$E_1$$ is globally asymptotically stable.

## Numerical analysis

In this section, we want to observe the dynamical behavior of our proposed model. In order to do this, we purpose numerical results by using Runge-Kutta of order 4th scheme which have used several authors for a wide range of problems consisting of ordinary differential equations. For the simulation purpose, we use different value of parameters used in the proposed model are given in the Table 1. In the set of parameters some are taken from published articles, while some of the parameters are taken in such a way, that would be much more biologically feasible. Furthermore the time interval is taken 50 months with initial population for susceptible, latent, acute infected, chronically infected, carrier, recovered and vaccinated individuals as 100, 80, 60, 40, 20, 0 and 20, respectively.

**Fig. 1:**
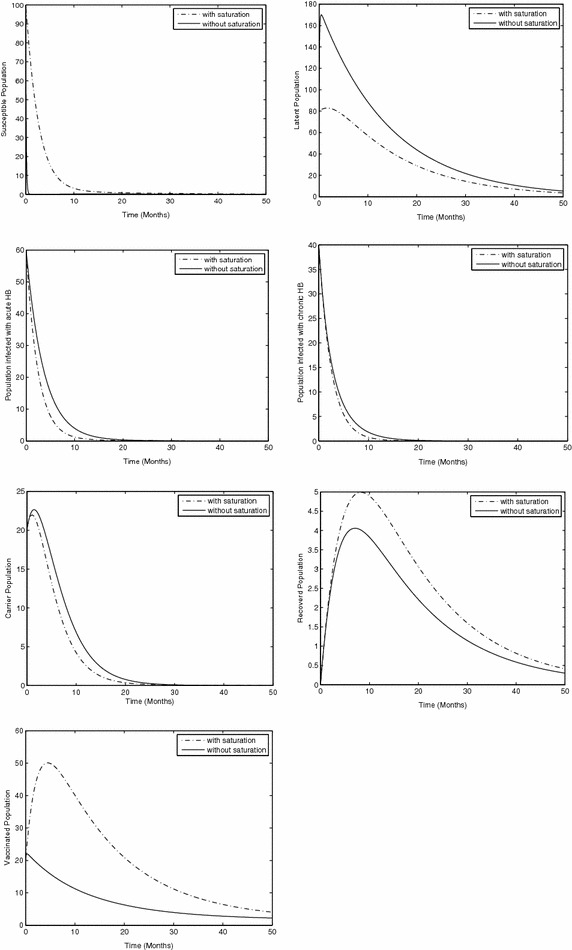
The plot shows the dynamical behavior of the proposed model (1) with saturation and without saturation

In the study of biological dynamic especially in the transmission dynamic of infectious disease sensitivity analysis is very important. Because by sensitive analysis, we are able to judge the role of every parameter and thus easily, we are in the position to develop a strategy for eradicating the disease from the community. Here we carry out a sensitivity analysis of our proposed model (1) with respect to the parameter $\alpha ,$ which are presented in Fig. 1.

In Fig. 1, the sensitivity analysis of susceptible, latent, acute infected with Hepatitis B, chronic infected with Hepatitis B, carrier, recovered and vaccinated are presented, which shows that, susceptible, recovered and vaccinated population are directly proportional to saturation $\alpha ,$ while latent, acute infected, chronically infected and carrier population are inversely proportional to saturation $\alpha .$ So from this brief analysis, it is observed that, saturation is also one of the factor to prevent the spreading of Hepatitis B in the world, which is one of the top three infectious diseases. Almost one third of the population are infected with Hepatitis B.

**Fig. 2:**
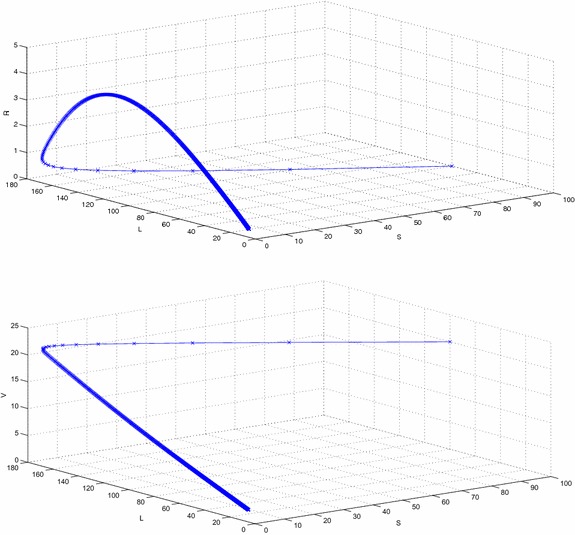
The plot shows the phase space diagram of susceptible, latent, recovered and susceptible, latent and vaccinated with stable endemic equilibrium $F_1$ and respective set of parameters given in Table 1

**Fig. 3:**
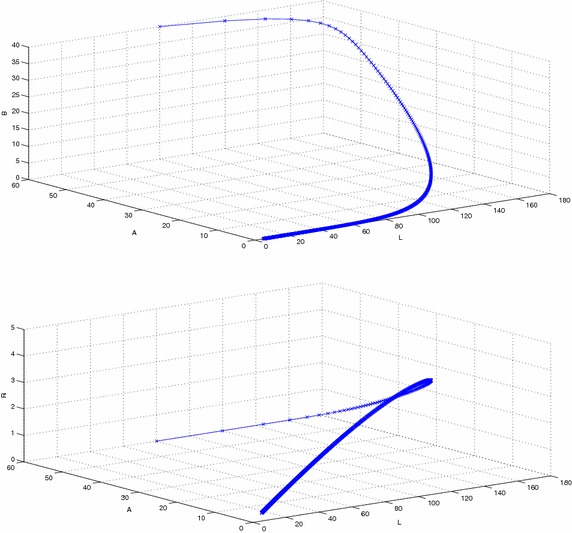
The plot shows the phase space diagram of latent, acute infected, chronically infected and latent, acute infected, recovered with stable endemic equilibrium $F_1$ and respective set of parameters given in Table 1

**Fig. 4:**
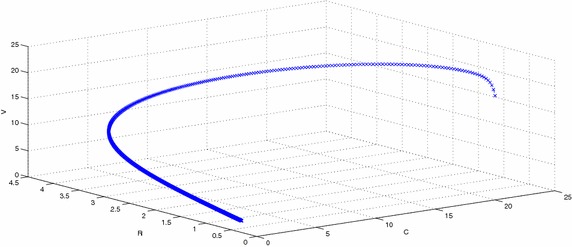
The plot shows the phase space diagram of carrier, recovered and vaccinated with stable endemic equilibrium $F_1$ and respective set of parameters given in Table 1

**Fig. 5:**
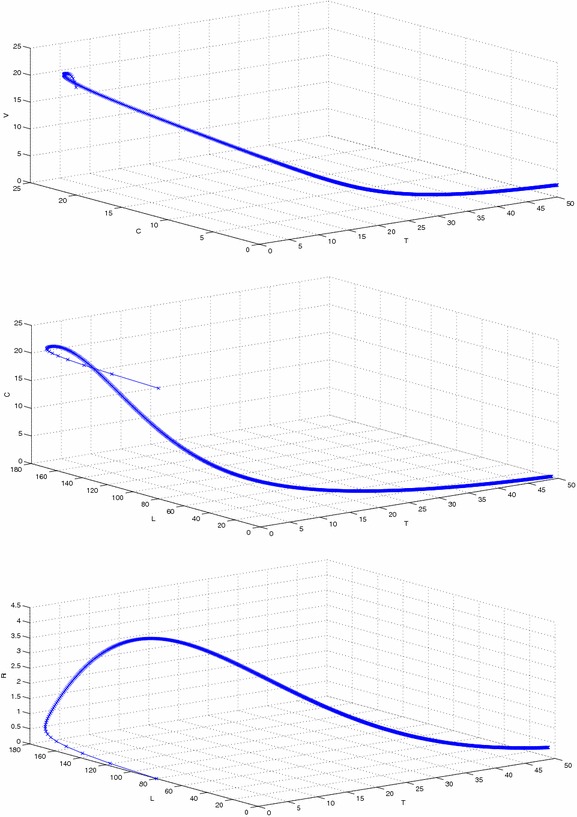
The plot shows the phase space diagram of time, susceptible, vaccinated, time, latent, carrier and time, latent, recovered with stable endemic equilibrium $F_1$ and respective set of parameters given in Table 1

Figure 2 represents the phase space diagram of susceptible, latent, recovered and susceptible, latent, vaccinated with stable endemic equilibrium and respective set of parameters given in Table  1. Figure 3 represents the phase space diagram of latent, acute, chronically infected and latent, acute, recovered. Similarly Figs. 4 and  5 represents the phase space diagram of carrier, recovered, vaccinated, time, recovered, vaccinated, time, latent, carrier, time, latent and recovered respectively with stable endemic equilibrium and respective set of parameters given in Table 1.

**Table 1:** The value of parameters used for numerical simulation

Notation	Parameter description	Value	Source
*b*	Birth rate	0.0121	Zou et al. (2010)
$\xi$	Birth rate without successful vaccination	0.05	Assumed
$\eta$	Perintally infected individuals rate	0.11	Zou et al. (2010)
$\phi$	Wanning vaccine induced immunity rate	0.01	Assumed
$\beta$	Transmission rate from susceptible to infected	0.95	Zou et al. (2010)
$\gamma$	Reduced transmission rate	0.12	Assumed
$d_0$	Natural mortality rate	0.0069	Assumed
$\gamma _3$	Vaccination rate	0.9	Assumed
$\sigma$	Moving rate from latent to acute	0.0012	Assumed
$\gamma _1$	Moving rate from acute to chronic carrier	0.33	Assumed
$\gamma _2$	Moving rate from chronic carrier to immune	0.09	Assumed
$d_1$	Hepatitis B related death rate	0.00054	Assumed
*p*	Probability of those individuals, who fails to recover in acute class	0.885	Zou et al. (2010)
$\alpha$	Saturation	0–0.9	Assumed

## Conclusion

The contagious disease of Hepatitis B is one of the topmost three infectious diseases reported by WHO and the incidence rate is precise significant in the study of epidemic models. In this article, we developed an epidemic problem for the spreading dynamic of Hepatitis B virus by classification of different subclasses with saturated occurrence rate. This occurrence rate is more generalized from than bilinear incidence rate and seems more realistic. Therefore we divided the host population into seven epidemiological subclasses, namely, susceptible, latent, acute infected with Hepatitis B, chronically infected with Hepatitis B, carrier, recovered and vaccinated, then formulated the model with this new features. First, we find the basic reproduction number $R_0.$ As in epidemiological models, the mathematical models has two steady states, infected and noninfected steady states. So we obtained the two non-negative equilibria that is disease free $F_0$, which is always exist and locally stable for $R_0<1$ and endemic equilibria $F_1$, which exist only, if $R_0>1$, therefore locally stable, if $R_0>1$. This portents occur, since totally the flows along the axes of susceptible, vaccinated and recovered are always attractor to the point $F_0$, but the flow along the axes of the infected compartment that is latent, infected with acute, chronic Hepatitis B and carrier depend on the value of $R_0$. So if $R_0<1$, then the axes of infected compartments are attractor towards the illness free equilibrium $F_0$. But when the $R_0$ acroses one that is $R_0>1$, the axes of these compartment do not attract the disease free equilibrium $F_0$ and repels from it. Further more to show the global stability at $F_0$, we established the Lyapnovo function, while to to prove the global stability at $F_1$, we used the geometrical approach. Finally numerics of the proposed model are retrieved out to show the feasibility of the model.
